# Verification of Surface Dose for Flattening Filter and Flattening Filter Free Beams in Beam-Matched Medical Linear Accelerators

**DOI:** 10.31557/APJCP.2021.22.8.2577

**Published:** 2021-08

**Authors:** N Sushma, Shanmukhappa Kaginelli, Sathiaraj Palanivel, K M Ganesh

**Affiliations:** 1 *Department of Radiation Physics, Kidwai Memorial Institute of Oncology, Bengaluru, India. *; 2 *Division of Medical Physics, JSS Academy of Higher Education and Research, Mysuru, India. *

**Keywords:** Surface dose, parallel plate chamber, percentage depth dose, source to surface distance

## Abstract

**Background:**

The purpose of this study was to evaluate the surface dose (SD) of 6 and 10 MV flattening filter beam (FF) and flattening filter free (FFF) beam for different square field sizes in three Beam-matched medical linear accelerators using a parallel-plate ionization chamber.

**Materials and Methods:**

The experiment was carried out in a phantom composed of 40×40 cm^2^ solid Water slabs of varying thickness. Further sheets of solid water phantom were added to take readings in the build-up region for both SSD and SAD technique. Surface doses are measured with a PPC-05 chamber and DOSE 1 electrometer, at measurement depth of 1 mm interval and all results are plotted relative to the dose measured at D_max_ for various field sizes. Surface dose readings are therefore reported as relative surface dose.

**Results:**

Surface dose increased linearly with field size for both FF and FFF photon beams in all three beam-matched linear accelerators in both SSD and SAD setup. The surface dose of FFF was higher than FF beams in all field sizes. For the given energy the surface dose difference (relative to 10x10 cm^2^ field size of 6FF) between FF and FFF beam was larger for large field size. For 6FF and 6FFF beam the surface dose difference for 5x5 cm^2^ is -5.27%, and for 30x30 cm^2^ it is 12.91%. The measured surface dose differences between linear accelerators are not statically significant (P>0.989). Similarly, the surface dose difference between SSD and SAD setup was also analysed and had no statistical significance (P>0.849).

**Conclusion:**

Study showed that the surface dose difference between beam-matched linear accelerators are insignificant. The surface dose difference between SSD and SAD setup were also found negligible. Most importantly, changing patients between beam-matched linear accelerators will not have any significant changes in surface dose in clinical setup.

## Introduction

The ability to deliver the prescribed dose to the patient within a narrow tolerance is crucial to the success of curative intended radiation therapy. Changes in tumour control probability and normal tissue complication probability of 10-20% and 20-30% respectively, can result from a 5% difference in the delivered dosage (Chetty et al., 2007). The fact that megavoltage (MV) photon beams have a skin-sparing effect is well known, but the exact magnitude depends on a variety of treatment parameters. Depending on the doses delivered, the basal skin layer will experience a number of problems ranging from mild (erythema/epilation) to severe (desquamation/necrosis) reactions. According to the ICRP and the ICRU, the skin depth prescribed for realistic dosage measurements is 0.07 cm, which refers to the skin’s interface between the epidermis and dermis layers (“Report 24, ICRU” 1976; Venselaar et al., 2001). At the same time, precise doses to targets near the surface (such as for head, neck, and breast treatments) are critical to avoid under dosing. The clinical use of flattening filter-free (FFF) radiotherapy is growing as accelerators such as the Versa HD (Elekta AB, Stockholm, Sweden) and True Beam (Varian Medical Systems, Palo Alto, CA) are introduced. While surface doses for traditional flattening filter beams (FF) have been studied under a variety of conditions(Carl and Vestergaard, 2000; Kim et al., 1998) but FFF beams have a shortage of data. Simple surface dose vs. field size variations in FFF beams have been reported by several authors(Cashmore, 2016; Kragl et al., 2009), but data on beam-matched linacs are lacking. Switching patients among available linacs can be very convenient and desirable in any high-volume/high-throughput clinical centre. It may be necessary due to various factors such as linac failure, an unexpectedly high patient load etc. Switching patients treated with a higher treatment modality with different linacs, requires necessary careful thought. Having beam-matched linacs will not only improve patient care versatility, but also reduce the social and economic impact of system downtime (Sjöström et al., 2009). In our center four beam-matched Elekta™ linear accelerators were installed, which consists of three Versa HD ™ with photon energies (6FF, 10FF,15FF, 6FFF and 10FFF) and one Infinity™ (6FF, 10FF and 15FF). All linear accelerators are having Agility TM head equipped with 80 pairs of multi-leaf collimators (MLC) with a width of 5mm at the machine isocenter. The linear accelerators are named as LA1, LA2 LA3 and LA4 (numbers are indicating the accelerators). LA1, LA2, and LA3 are Versa HD™ and LA4 is InfinityTM. For FFF beams LA3 was used as a reference machine for LA1 and LA2 whereas for FF beam LA4 was the reference for LA1, LA2 and LA3. Many researchers were reported about the performance of beam-matched linear accelerators (Kairn et al., 2015; Bhangle et al., 2011). Gagneur and Ezzell (2013) and Xu et al., (2019) reported the patient’s specific quality assurance of VMAT, SBRT/SRT for beam-matched linear accelerators. To the best of our knowledge, we could not find any study-related to the surface dose measurement for beam-matched linear accelerators.

In our study surface dose and build-up doses on three beam-matched linear accelerators were evaluated for varying field size, energy in both SSD and SAD techniques.

## Materials and Methods

Elekta’s photon beam factory matching allows Percentage Depth Dose at 10cm (D10) to be within 1% for the beam-matched linacs and any averaged point dose (average of the measurements over a 1 cm range from that point) within the region covering 80% of full width at half maximum (FWHM) shall be within a 2% difference as compared to the same points from profiles of other beam-matched linacs for beam profiles of 10x10cm2 and 30x30 cm2 field sizes (Sarkar et al., 2013). The D10 of LA4 was kept as reference for FF beams in other linacs viz. LA1, LA2 and LA3 within ±1%. Similarly, for FFF beam D10 of LA3 was kept as reference for other two FFF linear accelerators viz. LA1 and LA2 within ±1%. Table 1 shows the D10 values for four linear accelerators and percentage difference from the reference linear accelerators. 

The surface doses delivered by the FF and FFF beams were measured using a plane-parallel ionization chamber (PPC-05, IBA-Scanditronix, Germany) with DOSE 1 (IBA, Germany) electrometer in a solid water-equivalent phantom with adequate backscattering material. The solid water phantom has a physical density of 1.04 g/cm^3^. The PPC05 chamber has a “coin-shaped” sensitive volume with chamber outer diameter of 30.0 mm, height of 14.0 mm, sensitive volume (nominal) of 46.0 mm^3^, guard ring diameter of 17.8 mm and guard ring width of 3.4 mm. 

**Figure 1:**
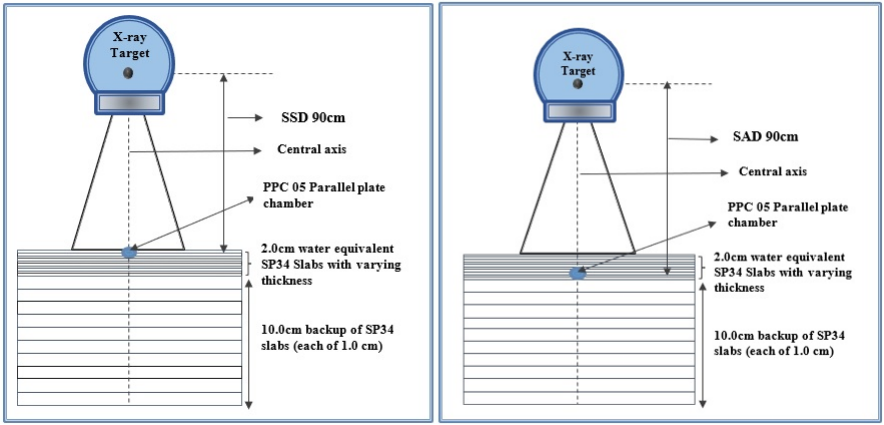
Schematic Diagram of the Measurement Setup Using a Plane‐Parallel Markus Chamber and a Stack of Water‐Equivalent Solid Phantom Slabs (Varying Thickness) for SSD and SAD Techniques

Figure 1 shows the experiment setup used to carry out measurement of surface dose in a phantom composed of 40 × 40 cm^2^ Solid Water slabs of varying thickness, with 10 cm of backscatter material to ensure full phantom scatter conditions. Further sheets of solid water phantom were added to take readings in the build-up region for both SSD and SAD technique. Surface doses are measured with a PPC-05 parallel plate ionization chamber and DOSE 1 electrometer. The chamber was embedded in the 2cm slab phantom such that the entrance window of the chamber was flush with the surface with the central axis perpendicular to it. Doses at depth were measured by adding layers of phantom material while maintaining the source to surface distance (SSD) at 90 cm to the top of the phantom. The effective point of measurement itself was taken as the inside of the entrance window which, for the PPC-05 chamber (with a composite window of 0.1 mm Mylar) is equivalent to 1 mm of water. Surface dose measurements therefore represent a measurement depth of 1 mm, and all results are plotted relative to the dose measured at Dmax for the field sizes of 5x5,10x10,15x15, 20x20 and 30x30 cm^2^. The measurement was repeated three times for an averaging purpose. Surface dose readings are therefore reported as relative surface dose (RSD) where RSD = Dsurface /Dmax. As the measurements were performed with PPC 05 chamber, to correct for over-response of chamber detector correction factor (Ci) is applied to the obtained surface dose values(Apipunyasopon et al., 2013). The correction factor “Ci (L)” is calculated using the empirical relation 

Ci (L) = ai (L)2 + bi (L) + di 

where Ci (L) is the correction (labelled by an index “i”) which is a function of the length of square field’s side (L) and ai, bi, and di are arbitrary constants that depend on the type of detector. For parallel plate chamber (Markus type), the constants were ai = −0.0006, bi = 0.0314, and di = 0.3628, and the obtained “Ci” values for the field sizes 5×5 cm^2^, 10×10 cm^2^, 15×15 cm^2^, 20×20 cm^2^, and 30×30 cm^2^ were 0.5048, 0.6168, 0.6988, 0.7508, and 0.7648, respectively. The procedure was repeated for all three beam-matched Versa HD linear accelerators for surface dose comparison. Data has been analysed for statistical significance using one-way ANOVA.

## Results

**Figure 2:**
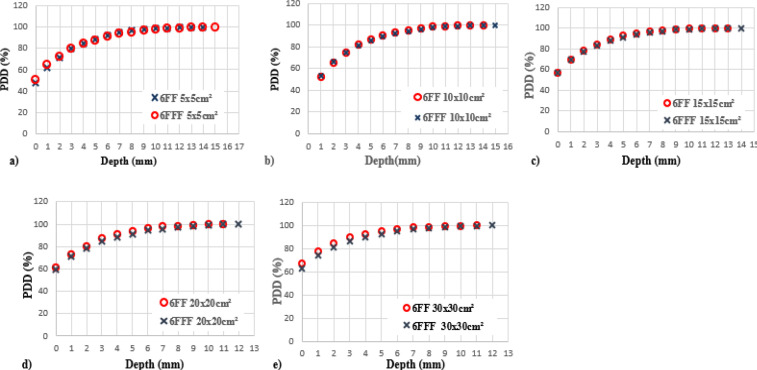
Measured Percentage Depth Dose (PDD) as a Function of the Depth (mm). Comparisons were made between flattening filter (FF) and flattening filter free (FFF) beams with a photon energy of 6 MV and five different field sizes of (a) 5x5 cm^2^, (b) 10x10 cm^2^, (c) 15x15 cm^2^, (d) 20x20 cm^2^ and (e) 30x30 cm^2^

Figure 2 depicts differences of the PDD between 6 FF and 6 FFF beams as a function of phantom depth at 1mm interval, for five various field sizes 5x5, 10x10, 15x15, 20x20 and 30x30 cm^2^. The same trend was observed for 10MV FF and FFF beams. Figure 3 (a,b,c and d) shows the percentage of surface dose of 6 and 10MV(FF andFFF) for SSD and SAD setup for a LA1, LA2 andLA3. From the figure it is very clear that the surface dose increases with the increase in field size. For all the linear accelerators, for both SSD and SAD setup there is a same trend observed in variations of surface dose as a function of field size in Figure 4 (a and b). FFF beams shows higher surface dose than FF beams for 5x5 and 10x10 cm^2^ field sizes. After 10x10 cm^2^, it was observed that there was an inverse in the trend, which means the FF beam surface dose was higher than the FFF beams. These trend variations observed for both 6 FF and 10FF beams. The surface dose difference of 6 FF and 6FFF was less compared to the difference of 10FF and10FFF (Figure 5). For given energy the surface dose difference (relative to 10x10cm^2^ field size of 6FF) between FF and FFF beam was larger for large field size. For 6FF and 6FFF beam the surface dose difference for 5x5 cm^2^ is -5.27%, and for 30x30 cm^2^ it is 12.91%. For 10FF and 10FFF beam the surface dose difference for 5x5cm2 is -9.8% ad for 30x30 cm^2^ it is 21.9%, all percentage difference was calculated relative to 10x10 cm^2^ of FF beams. 

In Table 2, it was found that the maximum surface dose for 6FFfor the reference field size of 10x10 cm^2^ is 32.99 ± 0.011 (LA3) in SSD setup and 32.57 ± 0.006 (LA1) in SAD setup. For 6FFF beam, the maximum surface dose for 10x10 cm^2^ is 34.03 ± 0.007 (SSD) and 33.37 ± 0.006 (SAD) for LA2 and LA1 respectively. For 10FF, the maximum surface dose was 26.86 ± 0.008 (SSD) and 26.78 ± 0.008 in LA1. For 10FFF beam the maximum surface dose 28.06 ± 0.002 (SSD) and 28.13 ± 0.007 (SAD) in LA3 and LA1 respectively. The maximum surface dose in maximum field size 30x30 cm^2^ for 6FF is 52.84 ± 0.011 (LA1) in SSD setup and 51.68 ± 0.005 (LA1) in SAD setup. For 6FFF the maximum surface dose was48.90 ± 0.022 (SSD) and 47.98 ± 0.006 (SAD) for LA2 and LA1 respectively. For 10FF, the maximum surface dose was 47.76 ± 0.023 (SSD) and 47.05 ± 0.006 in LA2 and LA1 respectively. For 10FFF beam the maximum surface dose 41.09 ± 0.047 (SSD) and 41.13 ± 0.008 (SAD) in LA3 and LA1 respectively.

In Table 2 and 3, the measured surface dose difference between linear accelerators are not significant statically (P>0.989). Similarly, the surface dose difference between SSD and SAD setup also revealed that they do not have any statistical significance (P>0.849).

**Table 1:** D10 Values for Four Linear Accelerators and Maximum Difference from the Reference Linear Accelerator

Energy (MV)	LA4 PDD (%)	LA3 PDD (%)	LA2 PDD (%)	LA1 PDD (%)	Maximum difference
6 FF	67.42	67.74	67.53	67.13	-0.47
10 FF	72.9	72.68	72.73	72.79	0.30
15 FF	76.36	76.28	75.93	75.79	0.78
6 FFF	NA	67.45	67.25	66.94	0.75
10 FFF	NA	72.79	72.53	72.44	0.48

**Table 2:** SD of 6 and 10 MV FF and FFF for Varying Field Sizes for SSD and SAD in All Three Linear Accelerators

Field size (cm²)	Energy (MV)	SSD			SAD		
		LA1	LA2	LA3	LA 1	LA 2	LA 3
		(SD %)	(SD %)	(SD %)	(SD %)	(SD %)	(SD %)
5X5	6 FF	24.24 ± 0.003	24.70 ± 0.005	25.31 ± 0.013	24.68 ± 0.006	24.44 ± 0.009	23.42 ± 0.010
	6 FFF	25.98 ± 0.007	26.30 ± 0.013	26.08 ± 0.012	25.85 ± 0.006	25.26 ± 0.010	24.81 ± 0.008
	10 FF	18.52 ± 0.012	18.62 ± 0.003	18.61 ± 0.005	19.09 ± 0.007	18.77 ± 0.029	17.78 ± 0.009
	10 FFF	21.15 ± 0.016	21.16 ± 0.013	21.14 ± 0.003	21.20 ± 0.034	20.98 ± 0.006	20.05 ± 0.051
10X10	6 FF	32.98 ± 0.011	32.88 ± 0.010	32.61 ± 0.104	32.57 ± 0.006	32.40 ± 0.008	31.51 ± 0.009
	6 FFF	33.50 ± 0.006	34.02 ± 0.007	33.56 ± 0.013	33.37 ± 0.006	33.15 ± 0.003	32.26 ± 0.004
	10 FF	26.85 ± 0.008	26.39 ± 0.009	26.48 ± 0.005	26.77 ± 0.008	26.47 ± 0.009	25.55 ± 0.006
	10 FFF	27.89 ± 0.019	27.98 ± 0.013	28.05 ± 0.002	28.13 ± 0.007	27.83 ± 0.016	26.91 ± 0.019
15X15	6 FF	40.76 ± 0.018	39.87 ± 0.010	40.28 ± 0.008	40.43 ± 0.006	40.14 ± 0.068	40.04 ± 0.013
	6 FFF	40.17 ± 0.006	40.82 ± 0.016	40.19 ± 0.068	39.97 ± 0.006	39.83 ± 0.003	39.45 ± 0.021
	10 FF	34.30 ± 0.021	34.01 ± 0.011	34.23 ± 0.020	34.74 ± 0.008	34.47 ± 0.005	34.19 ± 0.008
	10 FFF	33.70 ± 0.021	33.76 ± 0.014	33.68 ± 0.038	33.87 ± 0.008	33.64 ± 0.019	33.64 ± 0.010
20X20	6 FF	46.62 ± 0.011	46.64 ± 0.007	46.46 ± 0.013	46.56 ± 0.006	46.05 ± 0.022	46.11 ± 0.006
	6 FFF	45.38 ± 0.008	45.45 ± 0.008	45.75 ± 0.016	44.71 ± 0.006	44.47 ± 0.012	44.27 ± 0.007
	10 FF	40.83 ± 0.023	40.54 ± 0.005	40.86 ± 0.011	40.85 ± 0.007	40.57 ± 0.007	40.35 ± 0.008
	10 FFF	38.14 ± 0.023	37.89 ± 0.014	38.01 ± 0.005	38.22 ± 0.008	38.00 ± 0.082	37.68 ± 0.008
30X30	6 FF	52.83 ± 0.011	52.21 ± 0.024	51.37 ± 0.001	51.68 ± 0.005	51.37 ± 0.019	51.33 ± 0.005
	6 FFF	48.57 ± 0.052	48.90 ± 0.022	48.45 ± 0.011	47.98 ± 0.006	47.81 ± 0.007	47.60 ±0.006
	10 FF	46.70 ± 0.022	46.75 ± 0.023	46.45 ± 0.004	47.04 ± 0.006	46.85 ± 0.007	46.65 ± 0.006
	10 FFF	40.82 ± 0.024	40.73 ± 0.025	41.09 ± 0.047	41.12 ± 0.008	40.87 ± 0.004	40.65 ± 0.008

Values represent Surface Dose± Standard deviation; FF, Flattening Filter beam; FFF, Flattening Free Filter beam; LA1,LA2,LA3, Three linear accelerators

**Table 3:** Difference in SD of 6 and 10 MV FF and FFF for SSD and SAD in All Three Linear Accelerators

Field Size (cm²)	Energy (MV)	LA 1			LA 2			LA 3		
		SSD	SAD	Difference	SSD	SAD	Difference	SSD	SAD	Difference
		(SD %)	(SD %)		(SD %)	(SD %)		(SD %)	(SD %)	
5x5	6 FF	24.25	24.68	-0.018	24.7	24.45	0.01	25.32	23.42	0.075
	6 FFF	25.99	25.86	0.005	26.31	25.27	0.04	26.09	24.82	0.049
	10 FF	18.52	19.1	-0.031	18.62	18.78	-0.008	18.62	17.78	0.045
	10 FFF	21.16	21.21	-0.002	21.16	20.99	0.008	21.14	20.05	0.052
10x10	6 FF	32.99	32.57	0.013	32.89	32.4	0.015	32.62	31.51	0.034
	6 FFF	33.5	33.37	0.004	34.03	33.15	0.026	33.57	32.26	0.039
	10 FF	26.86	26.78	0.003	26.4	26.47	-0.003	26.48	25.55	0.035
	10 FFF	27.9	28.13	-0.008	27.98	27.83	0.005	28.06	26.91	0.041
15x15	6 FF	40.77	40.43	0.008	39.88	40.15	-0.007	40.29	40.04	0.006
	6 FFF	40.18	39.97	0.005	40.83	39.84	0.024	40.19	39.45	0.018
	10 FF	34.3	34.75	-0.013	34.02	34.47	-0.013	34.24	34.19	0.001
	10 FFF	33.7	33.87	-0.005	33.77	33.64	0.004	33.68	33.64	0.001
20x20	6 FF	46.63	46.56	0.001	46.64	46.05	0.013	46.46	46.11	0.008
	6 FFF	45.39	44.71	0.015	45.46	44.47	0.022	45.76	44.27	0.032
	10 FF	40.83	40.85	-0.001	40.55	40.58	-0.001	40.86	40.36	0.012
	10 FFF	38.15	38.22	-0.002	37.89	38.01	-0.003	38.01	37.68	0.009
30x30	6 FF	52.84	51.68	0.022	52.22	51.38	0.016	51.38	51.34	0.001
	6 FFF	48.58	47.98	0.012	48.9	47.81	0.022	48.46	47.6	0.018
	10 FF	46.71	47.05	-0.007	46.76	46.86	-0.002	46.46	46.66	-0.004
	10 FFF	40.82	41.13	-0.007	40.73	40.87	-0.003	41.09	40.65	0.011

Values represent surface dose (SD); SSD, Source to Surface Distane; SAD, Source to Axis Distance; LA1,LA2,LA3, Three linear accelerators.

**Figure 3a:**
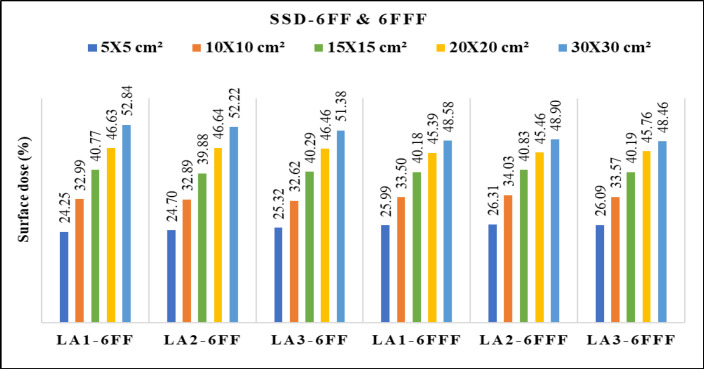
Variation of Surface Dose for 6 MV FF and FFF Beams Measured with SSD for Three Beam Matched Linear Accelerators

**Figure 3b:**
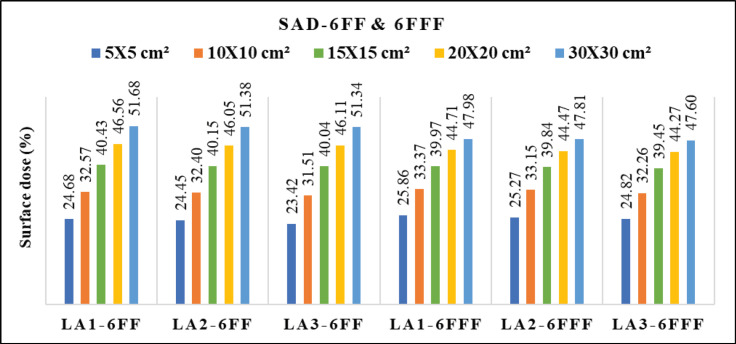
Variation of Surface Dose for 6 MV FF and FFF Beams Measured with SAD for Three Beam Matched Linear Accelerators

**Figure 3c:**
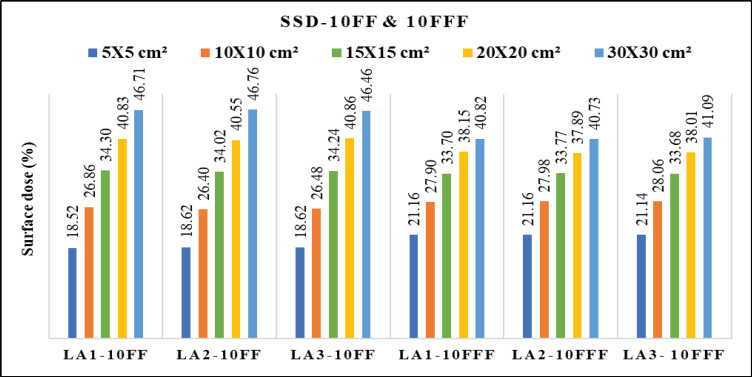
Variation of Surface Dose for 10 MV FF and FFF Beams Measured with SSD for Three Beam Matched Linear Accelerators

**Figure 3d:**
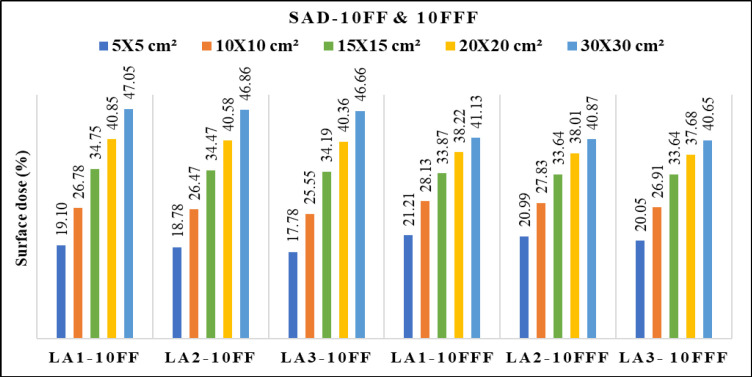
Variation of Surface Dose for 10 MV FF and FFF Beams Measured with SAD for Three Beam Matched Linear Accelerators

**Figure 4a:**
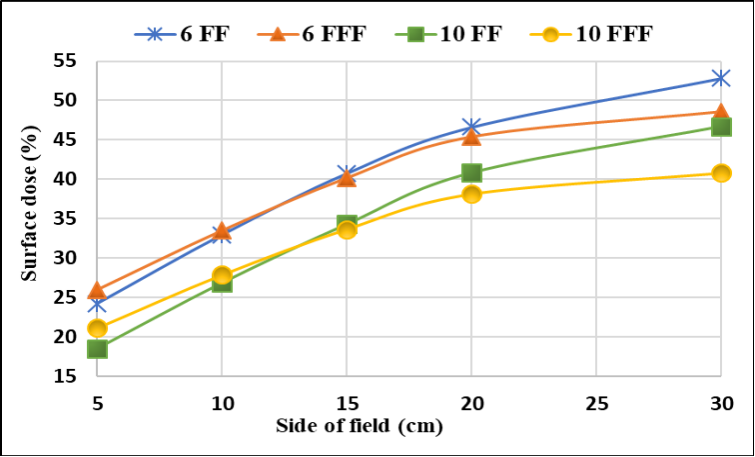
Variation of Surface Dose with Field Size for 6 and 10 MV FF and FFF Beams (SSD) in LA1

**Figure 4b:**
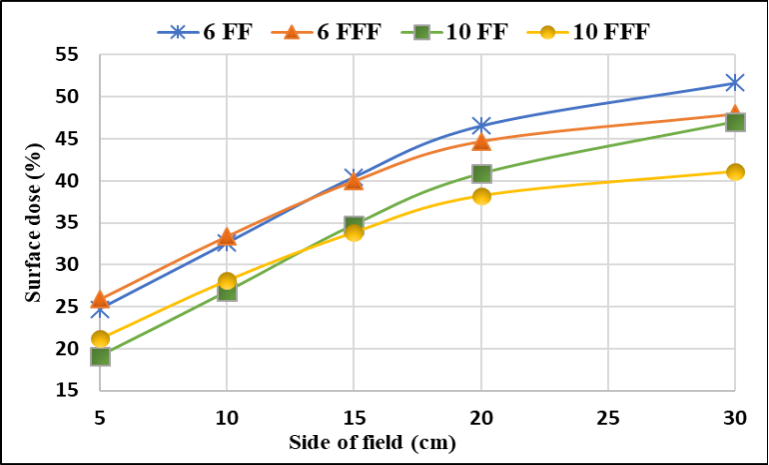
Variation of Surface Dose with Field Size for 6 and 10 MV FF and FFF Beams (SAD) in LA1

**Figure 5:**
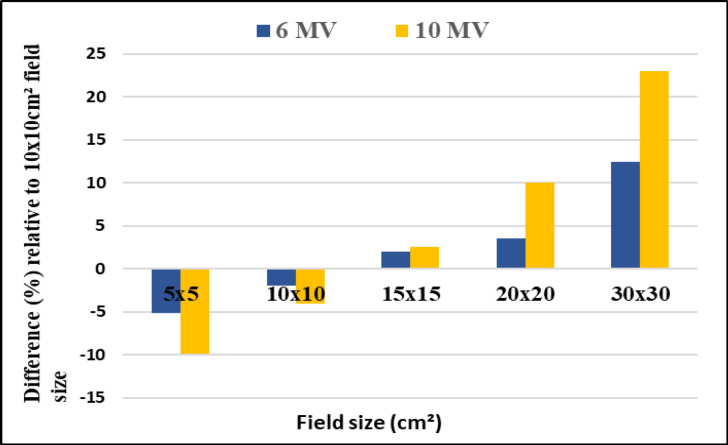
Relative Difference of SD with 10 x 10cm^2^ Field Size for 6 and 10 MV FF Beams

## Discussion

There are three major components which are contributing to surface dose such as head scatter, electron contamination and electron backscatter from the patients. All these factors arises due to beam energy, field size, source to surface distance, beam modifying devices (wedge, compensators and shadow trays) also contribute to the surface dose (Sigamani et al., 2016). In general, there is no model in treatment planning system accurately accounting for surface dose measurements. Huge difference between surface dose of TPS and measured dose is most commonly observed (Panettieri et al., 2009; Wang et al., 2018). Since, TPS estimation of surface dose is not much accurate and reliable, we need to rely on the physical measurement whenever the clinical situation is demanding. Surface dose measurements is more complex due to the dis-equilibrium behaviour of the beam in the surface/build-up region. As of now extrapolation chamber is the gold standard method for determining the surface reported dose (Reynolds and Higgins, 2015). In addition, with extrapolation chambers the feasibilities of surface dose measurements using other dosimeters such as fixed parallel plate ion chamber, film and cylindrical ion chambers also shown in the literature (Akbas et al., 2016). Eyadeh et al., (2017) used gel dosimeter for surface dose measurements, and they compared the results with film, results were comparable. Kinhikar et al., (2009)performed the surface dose measurements using MOSFET and TLD, they showed that inter-fraction deviation of surface dose was within 1.4%. Reynolds and Higgins, (2015) reported about surface dose measurements using TLD, film, OSLD and extrapolation chamber. They compared the dose with extrapolation chamber and they found that the closest match was obtained with the Attix chamber (−0.1%), followed by pTLD (0.5%), Capintec (4.5%), Memorial (7.3%), Markus (10%), cTLD (11.8%), eOSL (12.8%), EBT2 (14%), EDR2 (14.8%), and OSL (26%). The problem associated with fixed parallel plate ion chambers are overresponse in the build-up region. The imbalanced scattering between chamber cavity and medium cause overresponse of the chamber especially in the build-up region, this overresponse can be corrected by applying appropriate correction factors. Gerbi et al., reported the correction factors for correcting the over response of the chamber. 

Removing flattening filter increase the dose rate and reduces the extra field scatter. But it increases the low energy components in the spectrum and lead the increment in the surface dose. In this study both FF and FFF beams were analysed for surface dose measurements for different field size. It was observed that the smaller FFF fields were having higher surface dose than FF, beyond 15x15cm^2 ^the FF surface dose was higher. The same kind of trend have been noticed in the literature Lonski et al., (2017). 

From the Figure 3(a and b) one can understand that the FFF beam gives more surface dose for field size 5 x 5 cm^2 ^to 15x15cm^2^. Beyond 15 x 15cm^2^, FF beam gives higher surface dose than FFF for both 6 and 10 MV energies. This trend may be due to two reasons. Firstly, in a smaller field of FF beam, the collimator settings allow the radiation which is passing through from the central portion of the flattening filter and the concentration of primary radiation is more in such a small field. This primary beam has higher penetration power and this could cause less surface dose. Secondly, when the field size increases, the collimator opening allows more scatter radiation which is originating from the flattening filter (major part), jaws, MLCs, monitor chamber and will mix with the primary component of the beam and reduces the mean energy of radiation which are striking the surface, this softer beam may attribute for an increase in the surface dose. Similarly, in case of FFF beam, the main scattering source of radiation (flattening filter) is absent and the beam may not be harder compared to FF, such a softer FFF beam in a small field causes more surface dose than FF. As the field size increases the scattering components in FFF beam may not increase as like in FF, due to less scattering component in FFF, hence it produces less surface dose in larger field sizes. In short, one can interpret that the FF beam is softer than the FFF for larger field size, and for smaller field size FF beam is harder than the FFF beam. 

More than one linear accelerator in the institution can be tuned in order to produce same dosimetric quantities called beam-matched linear accelerators. Though, it is beam-matched it should be validated by the physicist before clinical application. This study approached the beam-matched linear accelerators in terms of surface dose measurements, though there is no control over surface dose of beam-matched linear accelerators, it should be verified as a clinical part. Though, linear accelerators are beam-matched, there could be a slight spectral variation between them. This may affect the dosimetric quantities, this study has been done by assuming that the inherent spectral variation cause deviations in surface dose. This study demonstrated that the surface dose is not varying significantly between the beam-matched linear accelerators. Many limitations are there in this study such as, a) except parallel plate ion chamber no other detector are used to confirm the reproducibility of the linear accelerators, b) Surface dose is an important concern in the TBI distance, that is not reported here, c) The results are not compared with any simulated data such as Monte Carlo simulations. Even though the study has above limitations, this is the first study aimed to report the surface dose measurements of beam-matched linear accelerators. 

In conclusion, the surface dose measurements have been done for beam-matched linear accelerator for FF and FFF beams in SSD and SAD setup. The FFF beam has higher surface dose up to 15x15 cm^2^. Beyond 15x15 cm^2^ FF beam has higher surface dose. The surface dose difference between SSD and SAD setup is not significant. Most importantly, study showed that the surface dose difference between beam-matched linear accelerators are insignificant. Changing patients between beam- matched linear accelerators will not have any significant changes in surface dose in clinical setup. This study could be further explored to report the response of surface dose at extended SSD. 

## Author Contribution Statement

All the authors have contributed substantially in the study.
